# Noninvasive Quantification of Hepatic Steatosis Using Ultrasound‐Derived Fat Fraction (CHESS2303): A Prospective Multicenter Study

**DOI:** 10.1002/mco2.70123

**Published:** 2025-02-27

**Authors:** Yunlin Huang, Jia Li, Chuan Liu, Danlei Song, Chuanlong Zhu, Yongfeng Ren, Jiaojian Lv, Longfeng Jiang, Rong Shan, Hao Wang, Zhou Wang, Siqin Long, Fan Jiang, Xiang Xie, Liren Lu, Ruixiang Qi, Pengfei Rong, Chuxiao Shao, Wang Yao, Youfang Gao, Wenping Wang, Juan Cheng, Vincent Wai‐Sun Wong, Ying Wang, Wai‐Kay Seto, Yi Dong, Christoph F. Dietrich, Xiaolong Qi

**Affiliations:** ^1^ Department of Ultrasound Xinhua Hospital Affiliated to Shanghai Jiao Tong University School of Medicine Shanghai China; ^2^ Department of Ultrasound Zhongda Hospital Medical School Southeast University Nanjing China; ^3^ Liver Disease Center of Integrated Traditional Chinese and Western Medicine Department of Radiology Zhongda Hospital Medical School Southeast University Nurturing Center of Jiangsu Province for State Laboratory of AI Imaging & Interventional Radiology (Southeast University) Nanjing China; ^4^ Basic Medicine Research and Innovation Center of Ministry of Education Zhongda Hospital, Southeast University State Key Laboratory of Digital Medical Engineering Nanjing China; ^5^ Department of Infectious Diseases The First Affiliated Hospital of Nanjing Medical University Nanjing China; ^6^ Department of Ultrasound Bozhou Hospital Affiliated to Anhui Medical University Bozhou China; ^7^ Department of Liver Disease Lishui People's Hospital Lishui China; ^8^ Department of Ultrasound Shandong Public Health Clinical Center Shandong University Shandong China; ^9^ Department of Ultrasound Medicine the Second Affiliated Hospital of Anhui Medical University Hefei China; ^10^ Department of Interventional Therapy The Second Affiliated Hospital of Anhui Medical University Hefei China; ^11^ Department of Ultrasound Affiliated Hangzhou First People's Hospital Zhejiang University School of Medicine Hangzhou China; ^12^ Department of Radiology The Third Xiangya Hospital of Central South University Changsha China; ^13^ Key Laboratory of Joint Diagnosis and Treatment of Chronic Liver Disease and Liver Cancer of Lishui Lishui People's Hospital Lishui China; ^14^ Department of Ultrasound Lishui People's Hospital Lishui China; ^15^ Department of Infectious Disease The People's Hospital of Bozhou Bozhou China; ^16^ Department of Ultrasound Zhongshan Hospital Fudan University Shanghai China; ^17^ Department of Medicine and Therapeutics The Chinese University of Hong Kong Hong Kong China; ^18^ Department of Medicine Queen Mary Hospital The University of Hong Kong Hong Kong China; ^19^ Department of Allgemeine Innere Medizin Kliniken Hirslanden Beau Site Salem Und Permanence Bern Switzerland

**Keywords:** hepatic steatosis, magnetic resonance imaging proton density fat fraction (MRI‐PDFF), noninvasive, quantification, ultrasound‐derived fat fraction (UDFF)

## Abstract

Ultrasound‐derived fat fraction (UDFF) is designed to assess the hepatic fat content quantitatively. A multicenter study that verifies the diagnostic performance of UDFF for detecting hepatic steatosis has not yet been reported. This study aimed to evaluate the performance of UDFF for diagnosing and grading hepatic steatosis. Participants referred for assessment of hepatic steatosis were prospectively recruited from eight hospitals. All participants underwent UDFF and magnetic resonance imaging proton density fat fraction (MRI‐PDFF) examinations. MRI‐PDFF was used as the reference for diagnosing hepatic steatosis. From January 2023 to July 2023, a total of 300 participants were included. The median body mass index was 25.4 kg/m^2^ (interquartile range: 22.7–28.1). UDFF values were positively correlated with MRI‐PDFF (*R* = 0.80, *p* < 0.001). Using MRI‐PDFF ≥ 5%, ≥ 15%, and ≥ 25% as the reference standard for detecting mild, moderate, and severe hepatic steatosis, the best cutoff values of UDFF were 7.6% (area under the receiver operating characteristic curves [AUC] = 0.90), 15.9% (AUC = 0.90), and 22.3% (AUC = 0.91), respectively. Thus, UDFF has excellent diagnostic performance in detecting and grading hepatic steatosis.

## Graphical abstract

In this prospective multicenter study, UDFF values were positively correlated with MRI‐PDFF (*R* = 0.80). Taking MRI‐PDFF ≥ 5%, ≥ 15%, and ≥ 25% as the reference for diagnosing mild, moderate, and severe hepatic steatosis, the cutoff values of UDFF were 7.6%, 15.9%, and 22.3% in the training set (AUC = 0.90–0.91), respectively. UDFF achieved comparable AUCs in the validation set.

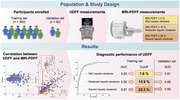

## 1 Introduction

Metabolic dysfunction associated steatotic liver disease (MASLD) [1] is the most common cause of chronic liver disease worldwide and affects approximately 30% of the adult population [2, 3]. MASLD is manifested as a range of hepatic complications and leads to progressive liver fibrosis, cirrhosis, liver failure, and hepatocellular carcinoma [4, 5]. There is a pressing need to develop simple and accurate noninvasive monitors to identify and stage the degree of hepatic steatosis.

Magnetic resonance imaging proton density fat fraction (MRI‐PDFF) is recommended as the reference standard for assessing hepatic steatosis and is increasingly used in clinical practice [6]. MRI‐PDFF reflects the tissue triacylglycerol concentration and is correlated well with histologic grade [7]. However, MRI‐PDFF examination is costly, and the use of MRI‐PDFF to monitor and follow up hepatic steatosis with increasing morbidity is not available.

Ultrasound is the first‐line examination in screening patients with fatty liver, as B mode ultrasound (BMUS) is readily available and low‐cost [6]. As the operator dependence, the reproducibility and accuracy of BMUS are limited [6]. Ultrasound‐based quantitative techniques for hepatic steatosis assessment have been designed to estimate the attenuation coefficient, backscatter coefficient, and speed of sound by utilizing radiofrequency signal data of ultrasound beam [6]. The attenuation coefficient refers to the decrease in ultrasound beam amplitude as it travels through tissue, while the backscatter coefficient measures the fraction of ultrasound energy reflected from tissue [8]. Based on the attenuation coefficient, the controlled attenuation parameter (CAP) enables the assessment of steatosis [6]. However, the liver morphological profile cannot be visualized during CAP measurement. The interpretation of CAP value requires knowledge of etiology [9], and there are no clear cutoff values of CAP to grade steatosis [10, 11]. Attenuation coefficient algorithms available on imaging ultrasound systems have been developed to evaluate hepatic steatosis. Still, there are some barriers to their widespread application in clinical practice, including the presentation of acquisition results and variation of measurement protocols among different ultrasound vendors [12]. The results of the ultrasound‐based quantitative technique, as presented as percentages, could improve clinical use.

Ultrasound‐derived fat fraction (UDFF) measurement has been designed to quantitatively assess the fat content of the liver based on the combination of attenuation coefficient and backscatter coefficient. UDFF integrates reference phantom data into the ultrasound system, eliminating the need for separate scans after each liver scan [6]. The UDFF value is presented as a percentage of hepatic steatosis, consistent with MRI‐PDFF. Although previous studies demonstrated good diagnostic performance of UDFF in assessing hepatic steatosis, these studies have been limited by small sample sizes, single‐center design, or hepatic steatosis evaluated using CAP or BMUS rather than MRI‐PDFF [13, 14, 15, 16]. A multicenter study that verifies the performance of UDFF for diagnosing and grading hepatic steatosis has not yet been reported.

Our study aimed to assess the role of UDFF measurement in detecting and quantifying hepatic steatosis using MRI‐PDFF as the reference standard.

## 2 Results

### 2.1 Participant Characteristics

**FIGURE 1:**
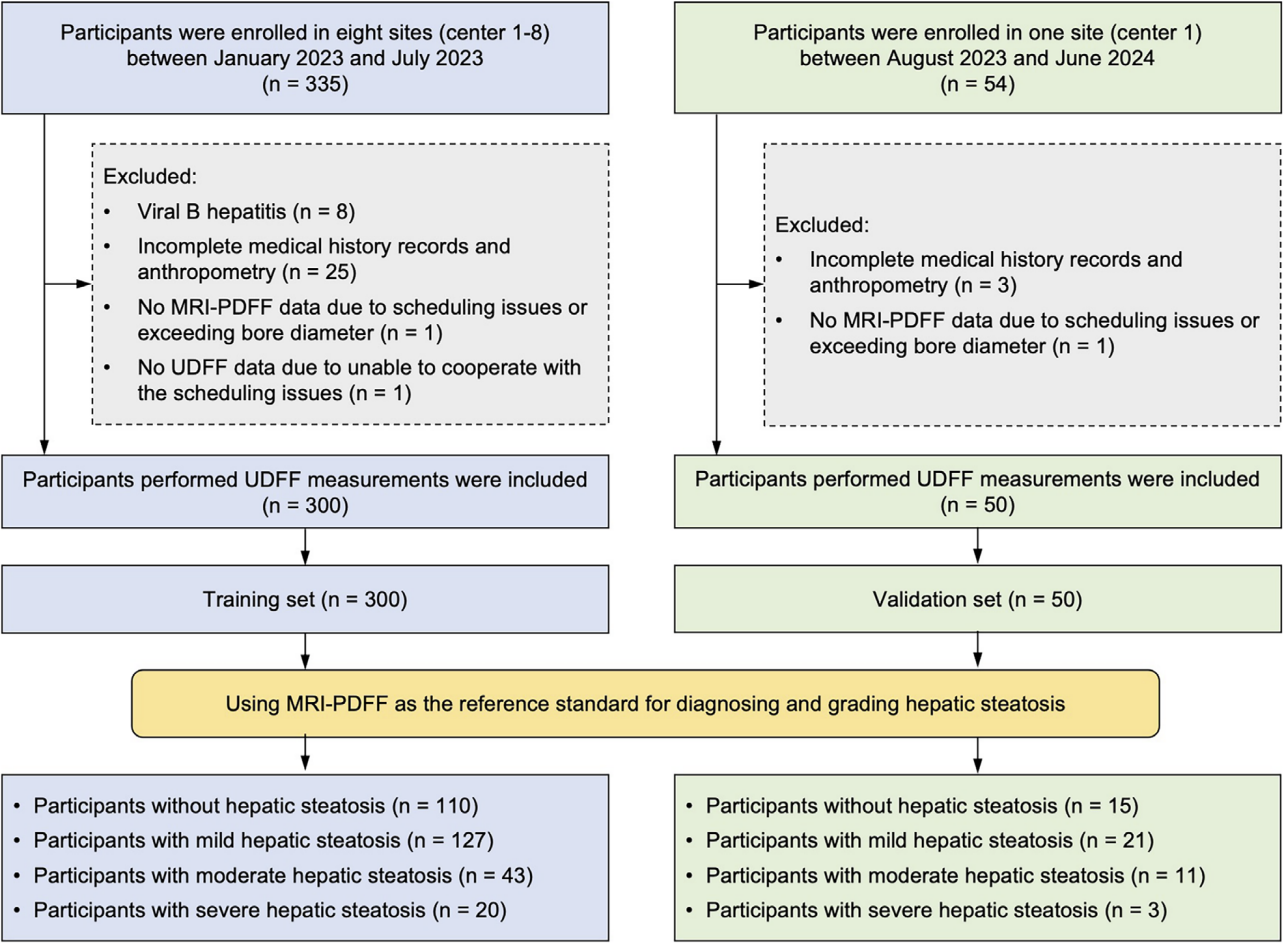
The flow diagram of the study cohort. UDFF, ultrasound‐derived fat fraction; MRI‐PDFF, magnetic resonance imaging proton density fat fraction.

Between January 2023 and July 2023, a total of 335 consecutive participants were prospectively recruited in the study. Thirty‐five participants were excluded due to viral B hepatitis (*n* = 8), incomplete medical history records and anthropometry (*n* = 25), exceeding bore diameter during MRI‐PDFF measurement (*n* = 1), and uncooperative during UDFF measurement (*n* = 1). Finally, 300 participants were included in the primary cohort as the training set. The flowchart of the study cohort is illustrated in Figure 1. Forty‐two participants were from Center 1, 125 were from Center 2, 14 were from Center 3, 20 were from Center 4, 25 were from Center 5, 30 were from Center 6, 20 were from Center 7, and 24 were from Center 8. The baseline characteristics of participants enrolled at each center are shown in Table S1.

In the training set, 51.0% (153/300) were male and 49.0% (147/300) were female. The median age and body mass index (BMI) were 39.0 years (interquartile range [IQR]: 30.0–52.0) and 25.4 kg/m^2^ (IQR: 22.7–28.1), respectively. Among the 300 participants, 36.3% (109/300) and 16.7% (50/300) were categorized as over‐weight (BMI: 25–30 kg/m^2^) and obese (BMI ≥ 30 kg/m^2^) [17], respectively; 45.3% (136/300) had dyslipidemia, 39.0% (117/300) had type 2 diabetes mellitus (T2DM), and 15.0% (45/300) had hypertension. The characteristics of the overall participants are detailed in Table 1. Taking MRI‐PDFF ≥ 5% as the reference standard for diagnosing hepatic steatosis, 190 (63.3%) participants had hepatic steatosis, whereas the remaining 110 (36.7%) participants were without hepatic steatosis. Participants with hepatic steatosis were more likely to be older males with a significantly higher prevalence of dyslipidemia, T2DM, and obesity (all *p* < 0.05). Moreover, almost all biochemical profiles were significantly higher in participants with hepatic steatosis than those without hepatic steatosis (all *p* < 0.05), except platelet, albumin, and total bilirubin. In addition, UDFF values (median and IQR) and skin‐to‐capsule distance on BMUS in participants with hepatic steatosis were significantly higher than those without hepatic steatosis (all *p* < 0.001). Noteworthy, no significant differences were observed regarding the quality indicator (IQR/Median) of UDFF and auto point shear wave elastography (shear wave velocity [SWV] and Young's modulus) in participants with hepatic steatosis compared to participants without hepatic steatosis (*p* > 0.05). Factors associated with hepatic steatosis were evaluated by univariate and multivariate analysis. T2DM, hypertension, BMI, alanine aminotransferase (ALT), UDFF, and skin‐to‐capsule distance on BMUS were determinant factors associated with hepatic steatosis (*p* < 0.05) (Figure S1).

**TABLE 1:** Characteristics of participants in the training set.

Characteristics	Participants with hepatic steatosis (*n* = 190)	Participants without hepatic steatosis (*n* = 110)	*p* value
**Demographic data**			
Age (years)	40.5 (33.0–53.0)	36.0 (25.0–49.0)	0.001
Sex			< 0.001
Male	120 (63.2)	33 (30.0)	
Female	70 (36.8)	77 (70.0)	
BMI (kg/m^2^)	27.1 (24.6–30.1)	22.5 (20.4–24.6)	< 0.001
**Metabolic factors**			
Dyslipidemia	118 (62.1)	18 (16.3)	< 0.001
T2DM	92 (48.4)	25 (22.7)	< 0.001
Hypertension	35 (18.4)	10 (9.1)	0.078
Obesity	49 (25.8)	1 (0.9)	< 0.001
**Biochemical profile**			
Platelet (10^9^/L)	232.0 (191.0–278.5)	224.0 (187.0–260.5)	0.179
TC (mmol/L)	4.9 (4.3–5.6)	4.5 (3.8–5.1)	< 0.001
TG (mmol/L)	1.7 (1.3–2.7)	0.9 (0.7–1.2)	< 0.001
HDL‐C (mmol/L)	1.1 (1.0–1.4)	1.5 (1.2–1.7)	< 0.001
LDL‐C (mmol/L)	3.0 (2.5–3.5)	2.6 (2.0–3.3)	< 0.001
ALT (IU/L)	27.0 (20.0–45.0)	16.5 (13.0–26.2)	< 0.001
AST (IU/L)	22.2 (18.8–31.0)	20.0 (15.5–24.1)	< 0.001
GGT (IU/L)	36.0 (22.0–55.8)	18.0 (11.0–30.1)	< 0.001
Albumin (g/L)	45.0 (43.1–47.7)	45.0 (43.4–47.0)	0.997
Total bilirubin (µmol/L)	10.6 (8.8–14.1)	11.1 (8.7–13.5)	0.733
FPG (mmol/L)	5.7 (5.0–7.6)	5.0 (4.6–5.4)	< 0.001
HbA1c (%)	5.9 (5.4–7.7)	5.3 (4.8–5.5)	< 0.001
**Ultrasonic data**			
Median of UDFF (%)	14.7 (9.3–21.1)	4.3 (3.2–6.6)	< 0.001
IQR of UDFF (%)	1.8 (1.0–2.8)	0.8 (0.0–1.1)	< 0.001
IQR/median of UDFF	0.12 (0.07–0.21)	0.15 (0.00–0.24)	0.686
SWV (m/s)	1.13 (1.02–1.23)	1.12 (1.02–1.20)	0.433
Young's modulus (kPa)	3.86 (3.19–4.74)	3.82 (3.38–4.42)	0.802
Skin‐to‐capsule distance on BMUS (cm)	2.3 (2.0–2.7)	1.9 (1.6–2.5)	< 0.001
**MRI‐PDFF (%)**	11.6 (8.0–18.0)	3.1 (2.4–3.9)	< 0.001

*Note*: Quantitative variables were presented as median (interquartile range); qualitative variables were presented as absolute (number), and data in parentheses are the percentage (%); hepatic steatosis is defined as magnetic resonance imaging proton density fat fraction (MRI‐PDFF) ≥ 5%.

Abbreviations: ALT, alanine aminotransferase; AST, aspartate aminotransferase; BMI, body mass index; BMUS, B mode ultrasound; FPG, fasting plasma glucose; GGT, gamma‐glutamyl transferase; HbA1c, hemoglobin A1c; HDL‐C, high‐density lipoprotein cholesterol; IQR, interquartile range; LDL‐C, low‐density lipoprotein cholesterol; SWV, shear wave velocity; T2DM, type 2 diabetes mellitus; TC, total cholesterol; TG, triglycerides; UDFF, ultrasound‐derived fat fraction.

Taking MRI‐PDFF ≥ 5%, ≥ 15%, and ≥ 25% as the reference standard for grading steatosis, 36.7% (110/300) participants had no hepatic steatosis, 42.3% (127/300) participants had mild hepatic steatosis, 14.3% (43/300) participants had moderate hepatic steatosis, and 6.7% (20/300) participants had severe hepatic steatosis. The median MRI‐PDFF values of participants with mild, moderate, and severe hepatic steatosis were 8.9% (IQR: 6.9–11.6), 18.8% (IQR: 17.0–21.3), and 30.5% (IQR: 27.9–32.2), respectively.

### 2.2 Correlation Between UDFF and MRI‐PDFF

All UDFF measurements were successful, and no adverse events were observed. The median time to complete 6 UDFF acquisitions for each participant was 2.0 min (IQR: 2.0–3.0). The 6 actual individual UDFF measurements per participant are shown in Figure S2. The median UDFF of overall participants was 10.0% (IQR: 4.7–17.7).

**FIGURE 2:**
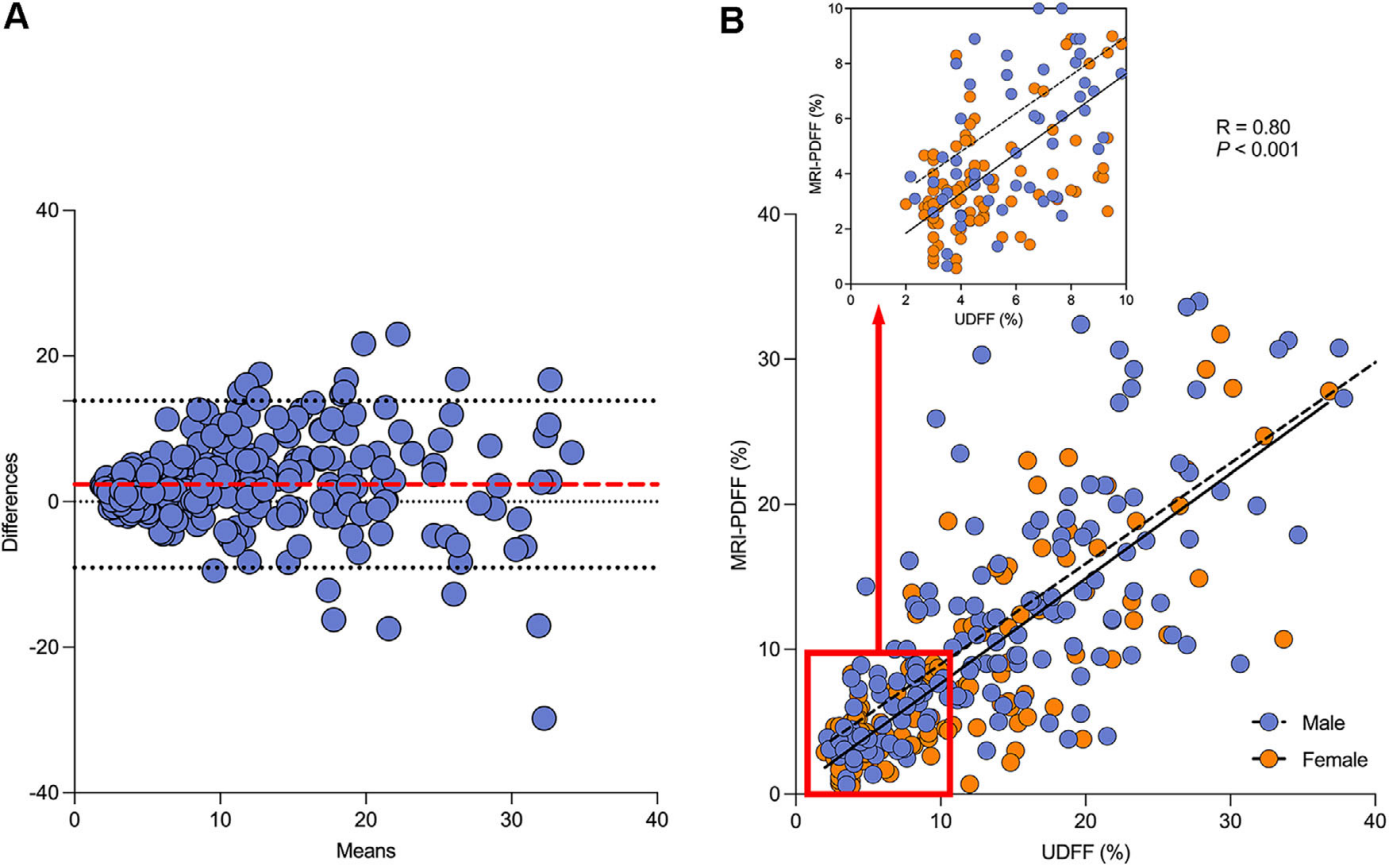
The correlation between ultrasound‐derived fat fraction (UDFF) and magnetic resonance imaging proton density fat fraction (MRI‐PDFF). (A) Bland–Altman plot shows that the mean difference in the liver fat content measured by UDFF and MRI‐PDFF is 2.36 (red dashed line), and the upper and lower 95% limits of agreement were 13.82 and −9.08, respectively (black dashed lines). (B) Scatterplots of UDFF versus MRI‐PDFF. UDFF was positively correlated with MRI‐PDFF, with a correlation coefficient of 0.80 (95% confidence interval: 0.75 to 0.84, *p* < 0.001).

The median interval between UDFF and MRI‐PDFF measurements was 1 day (IQR: 0–3). The Bland–Altman analysis showed the difference between UDFF and MRI‐PDFF values and the mean of the two types of values. The plot indicates that the bias, upper limit of agreement, and lower limit of agreement were 2.36, 13.82, and −9.08, respectively (Figure 2A). The UDFF was positively correlated with MRI‐PDFF, with a Spearman's correlation coefficient of 0.80 (95% confidence interval [CI]: 0.75 to 0.84, *p* < 0.001) (Figure 2B).

**FIGURE 3:**
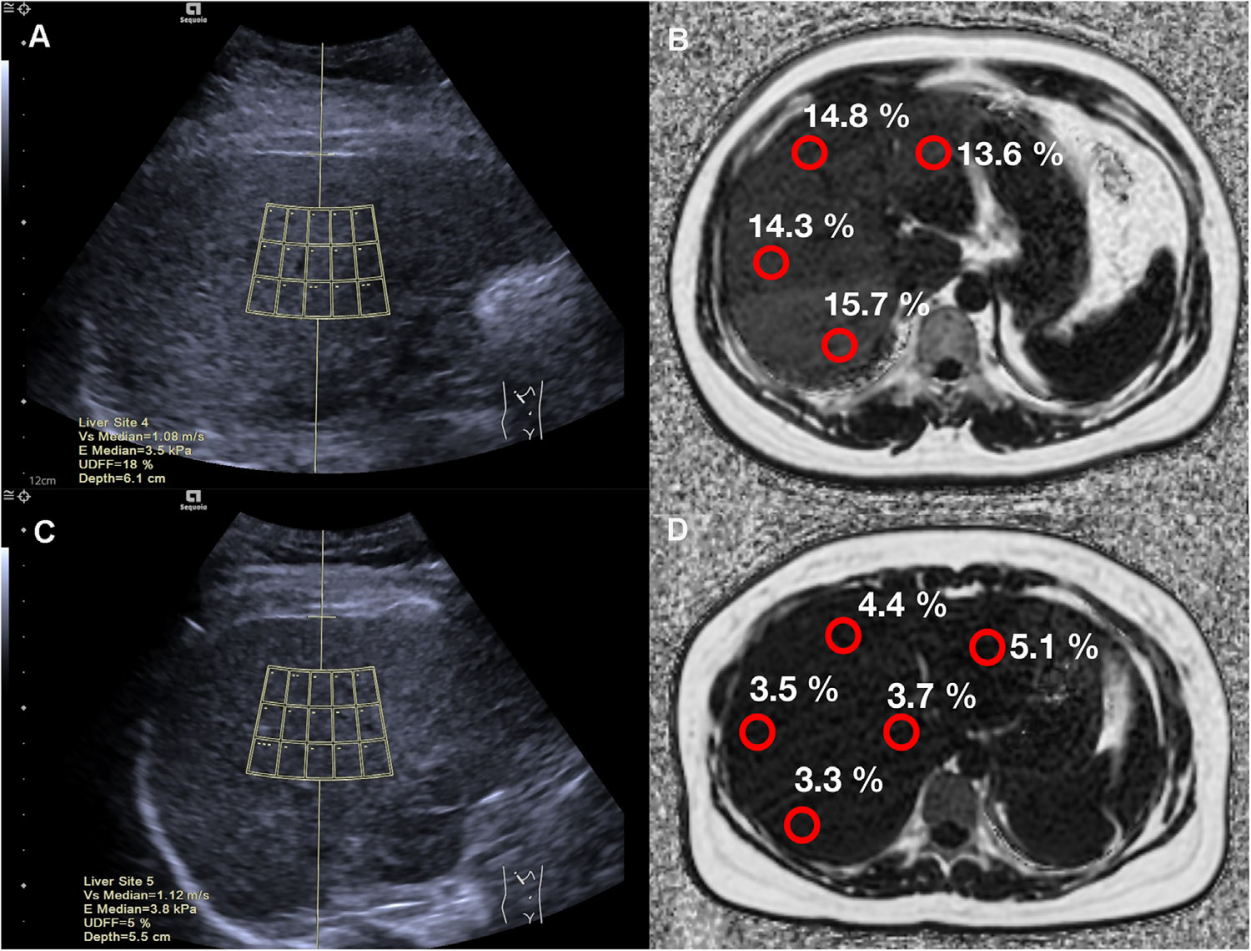
Representative images. (A) A 41‐year‐old female with a body mass index of 29.04 kg/m^2^ and an ultrasound‐derived fat fraction (UDFF) image displayed a value of 18%. (B) The magnetic resonance imaging proton density fat fraction (MRI‐PDFF) image showed a value of 14.8%. (C) A 45‐year‐old female with a body mass index of 24.74 kg/m^2^ and a UDFF image displayed a value of 5%. (D) The MRI‐PDFF image showed a value of 4.3%.

The median UDFF values of participants with mild, moderate, and severe hepatic steatosis were 11.8% (IQR: 8.2–16.2), 18.8% (IQR: 16.3–24.2), and 27.3% (IQR: 22.3–32.5), respectively. The UDFF values increased as the degree of hepatic steatosis increased. There was a significant difference in the UDFF values between mild, moderate, and severe steatosis (*p* < 0.05). The representative images of UDFF and MRI‐PDFF in participants with hepatic steatosis are shown in Figure 3A and Figure 3B, respectively. While the representative images of UDFF and MRI‐PDFF in participants without hepatic steatosis are shown in Figure 3C, D.

### 2.3 Diagnostic Performance of UDFF

**FIGURE 4:**
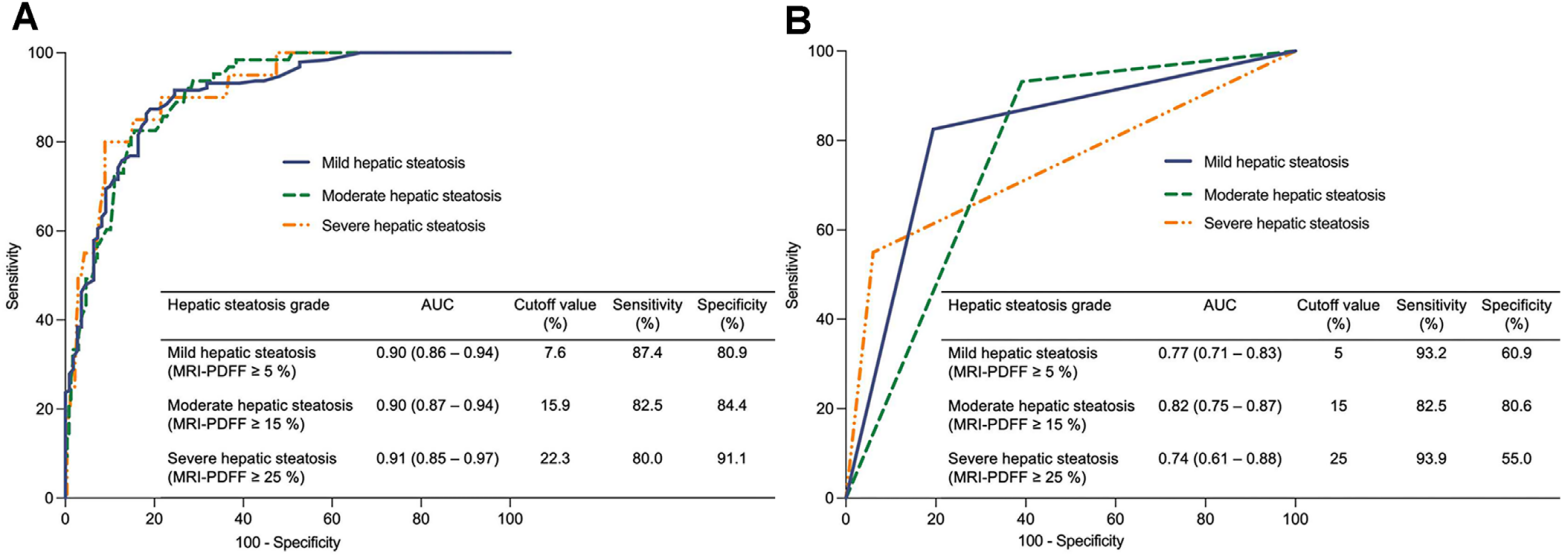
The area under the receiver operating characteristic curves (AUCs) of ultrasound‐derived fat fraction (UDFF) to diagnose hepatic steatosis in the training set. (A) Using magnetic resonance imaging proton density fat fraction (MRI‐PDFF) ≥ 5%, ≥ 15%, and ≥ 25% as the reference to detect mild, moderate, and severe hepatic steatosis, the AUCs of UDFF (cutoff values ≥ 7.6%, ≥ 15.9%, and ≥ 22.3%) were 0.90, 0.90, and 0.91, respectively. (B) Using the same threshold values as MRI‐PDFF, the AUCs of UDFF (cutoff values ≥ 5%, ≥ 15%, and ≥ 25%) for detecting mild, moderate, and severe hepatic steatosis were 0.77, 0.82, and 0.74, respectively.

In the training set, taking MRI‐PDFF ≥ 5% as a reference to detect mild hepatic steatosis, the best cutoff value of UDFF was 7.6%, with an area under the receiver operating characteristic curve (AUC) of 0.90 (95% CI: 0.86 to 0.94), a sensitivity of 87.4% (95% CI: 81.9 to 91.4), and a specificity of 80.9% (95% CI: 72.6 to 87.2). Taking MRI‐PDFF ≥ 15% as a reference to detect moderate hepatic steatosis, the best cutoff value of UDFF was 15.9%, with an AUC of 0.90 (95% CI: 0.87 to 0.94), a sensitivity of 82.5% (95% CI: 71.4 to 90.0), and a specificity of 84.4% (95% CI: 79.2 to 88.4). Taking MRI‐PDFF ≥ 25% as a reference to detect severe hepatic steatosis, the best cutoff value of UDFF was 22.3%, with an AUC of 0.91 (95% CI: 0.85 to 0.97), a sensitivity of 80.0% (95% CI: 58.4 to 91.9), and a specificity of 91.1% (95% CI: 87.2 to 93.9) (Table 2 and Figure 4A).

**TABLE 2:** Diagnostic performance of UDFF for hepatic steatosis as assessed with the MRI‐PDFF in the training and validation sets.

	Training set	Validation set
**Mild hepatic steatosis (MRI‐PDFF ≥ 5%)**		
Cutoff value (%)	7.6	
AUC	0.90 (0.86–0.94)	0.81 (0.66–0.96)
Sensitivity (%)	87.4 (81.9–91.4)	88.6 (74.1–95.5)
Specificity (%)	80.9 (72.6–87.2)	73.3 (48.1–89.1)
**Moderate hepatic steatosis (MRI‐PDFF ≥ 15%)**		
Cutoff value (%)	15.9	
AUC	0.90 (0.87–0.94)	0.66 (0.49–0.84)
Sensitivity (%)	82.5 (71.4–90.0)	57.1 (32.6–78.6)
Specificity (%)	84.4 (79.2–88.4)	75.0 (58.9–86.3)
**Severe hepatic steatosis (MRI‐PDFF ≥ 25%)**		
Cutoff value (%)	22.3	
AUC	0.91 (0.85–0.97)	0.95 (0.88–1.00)
Sensitivity (%)	80.0 (58.4–91.9)	100.0 (43.9–100.0)
Specificity (%)	91.1 (87.2–93.9)	89.4 (77.4–95.4)

Abbreviations: AUC, area under the receiver operating characteristic curve; MRI‐PDFF, magnetic resonance imaging proton density fat fraction; UDFF, ultrasound‐derived fat fraction.

While using the same threshold of MRI‐PDFF, the AUC of UDFF ≥ 5% for diagnosing mild hepatic steatosis was 0.77 (95% CI: 0.71 to 0.83), with a sensitivity of 93.2% (95% CI: 88.7 to 95.9) and specificity of 60.9% (95% CI: 51.6 to 69.5); UDFF (cutoff value ≥ 15%) had an AUC of 0.82 (95% CI: 0.75 to 0.87) for diagnosing moderate hepatic steatosis, with a sensitivity of 82.5% (95% CI: 71.4 to 90.0) and specificity of 80.6% (95% CI: 75.1 to 85.1); the AUC of UDFF ≥ 25% for diagnosing severe hepatic steatosis was 0.74 (95% CI: 0.61 to 0.88), with a high specificity of 93.9% (95% CI: 90.5 to 96.2) but low sensitivity of 55.0% (95% CI: 34.2 to 74.2) (Figure 4B).

### 2.4 Temporal External Validation

From August 2023 to June 2024, 50 participants meeting the same inclusion and exclusion criteria were exclusively extracted from one of the eight centers (center 1) for external validation (Figure 1). The validation set also consisted mainly of males (58.0%, 29/50) with a median age of 38.0 years (IQR: 33.8–42.3). The validation set had significantly higher BMI, platelet account, SWV, Young's modulus, and skin‐to‐capsule distance on BMUS than the training set (*p* < 0.001, *p* = 0.002, *p* = 0.002, *p* = 0.008, and *p* < 0.001, respectively). In the validation set, 30.0% (15/50) of participants were without hepatic steatosis, 42.0% (21/50) had mild hepatic steatosis, 22.0% (11/50) had moderate hepatic steatosis, and 6.0% (3/50) had severe hepatic steatosis (Table 3). The participants with mild, moderate, and severe hepatic steatosis had higher median UDFF values (14.0%, 15.5%, and 28.5%, respectively) than that of participants without hepatic steatosis (5.0%) (*p* = 0.001, *p* < 0.001, *p* = 0.001, respectively). However, no significant differences were found among participants with mild, moderate, and severe hepatic steatosis (all *p* > 0.05).

**TABLE 3:** Characteristics of the training and validation set.

Characteristics	Training set (*n* = 300)	Validation set (*n* = 50)	*p* value
**Demographic data**			
Age (years)	39.0 (30.0–52.0)	38.0 (33.8–42.3)	0.269
Male	153 (51.0)	29 (58.0)	0.359
BMI (kg/m^2^)	25.4 (22.7–28.1)	28.0 (26.0–29.3)	< 0.001
**Metabolic factors**			
Dyslipidemia	136 (45.3)	28 (56.0)	0.162
T2DM	117 (39.0)	24 (48.0)	0.230
Hypertension	45 (15.0)	2 (4.0)	0.059
Obesity	50 (16.7)	9 (18.0)	0.816
**Biochemical profile**			
Platelet (10^9^/L)	230.0 (188.3–272.8)	245.0 (224.0–291.0)	0.002
TC (mmol/L)	4.7 (4.1–5.5)	4.7 (4.2–5.6)	0.995
TG (mmol/L)	1.4 (0.9–2.1)	1.3 (0.9–1.9)	0.596
HDL‐C (mmol/L)	1.2 (1.0–1.5)	1.1 (1.0–1.4)	0.112
LDL‐C (mmol/L)	2.9 (2.3–3.5)	3.0 (2.6–3.5)	0.081
ALT (IU/L)	24.0 (16.0–37.8)	27.5 (19.8–40.8)	0.142
AST (IU/L)	21.0 (18.0–27.0)	21.5 (18.0–33.3)	0.761
GGT (IU/L)	27.0 (17.0–50.5)	28.5 (19.5–52.8)	0.630
Albumin (g/L)	45.0 (43.4–47.5)	45.5 (44.4–47.3)	0.277
Total bilirubin (µmol/L)	10.8 (8.8–14.1)	9.9 (8.4–14.9)	0.546
FPG (mmol/L)	5.3 (4.9–6.6)	5.3 (5.0–5.6)	0.685
HbA1c (%)	5.6 (5.2–6.5)	5.5 (5.2–5.8)	0.057
**Ultrasonic data**			
UDFF (%)	10.0 (4.7–17.7)	12.3 (6.5–17.1)	0.283
SWV (m/s)	1.12 (1.02–1.22)	1.30 (1.04–1.47)	0.002
Young's modulus (kPa)	3.84 (3.26–4.56)	5.05 (3.20–6.50)	0.008
Skin‐to‐capsule distance on BMUS (cm)	2.1 (1.7–2.7)	2.7 (2.4–2.9)	< 0.001
**MRI‐PDFF**			
MRI‐PDFF (%)	7.3 (3.7–13.3)	8.2 (4.6–16.3)	0.231
Normal liver (MRI‐PDFF < 5%)	110 (36.7)	15 (30.0)	0.362
Mild hepatic steatosis (MRI‐PDFF ≥ 5%)	127 (42.3)	21 (42.0)	0.965
Moderate hepatic steatosis (MRI‐PDFF ≥ 15%)	43 (14.3)	11 (22.0)	0.165
Severe hepatic steatosis (MRI‐PDFF ≥ 25%)	20 (6.7)	3 (6.0)	1.000

*Note*: Quantitative variables were presented as median (interquartile range). Qualitative variables were presented as absolute (number), and data in parentheses are the percentage (%).

Abbreviations: ALT, alanine aminotransferase; AST, aspartate aminotransferase; BMI, body mass index; BMUS, B mode ultrasound; FPG, fasting plasma glucose; GGT, gamma‐glutamyl transferase; HbA1c, hemoglobin A1c; HDL‐C, high‐density lipoprotein cholesterol; LDL‐C, low‐density lipoprotein cholesterol; MRI‐PDFF, magnetic resonance imaging proton density fat fraction; SWV, shear wave velocity; T2DM, type 2 diabetes mellitus; TC, total cholesterol; TG, triglycerides; UDFF, ultrasound‐derived fat fraction.

The diagnostic performance of UDFF for detecting mild and severe hepatic steatosis in the validation set achieved comparable AUCs to that of the training set (AUC = 0.81–0.95). However, the AUC of UDFF for diagnosing moderate hepatic steatosis was lower in the validation set (AUC = 0.66) than in the training set (AUC = 0.90) (Table 2).

### 2.5 Determinant Factors of UDFF

**FIGURE 5:**
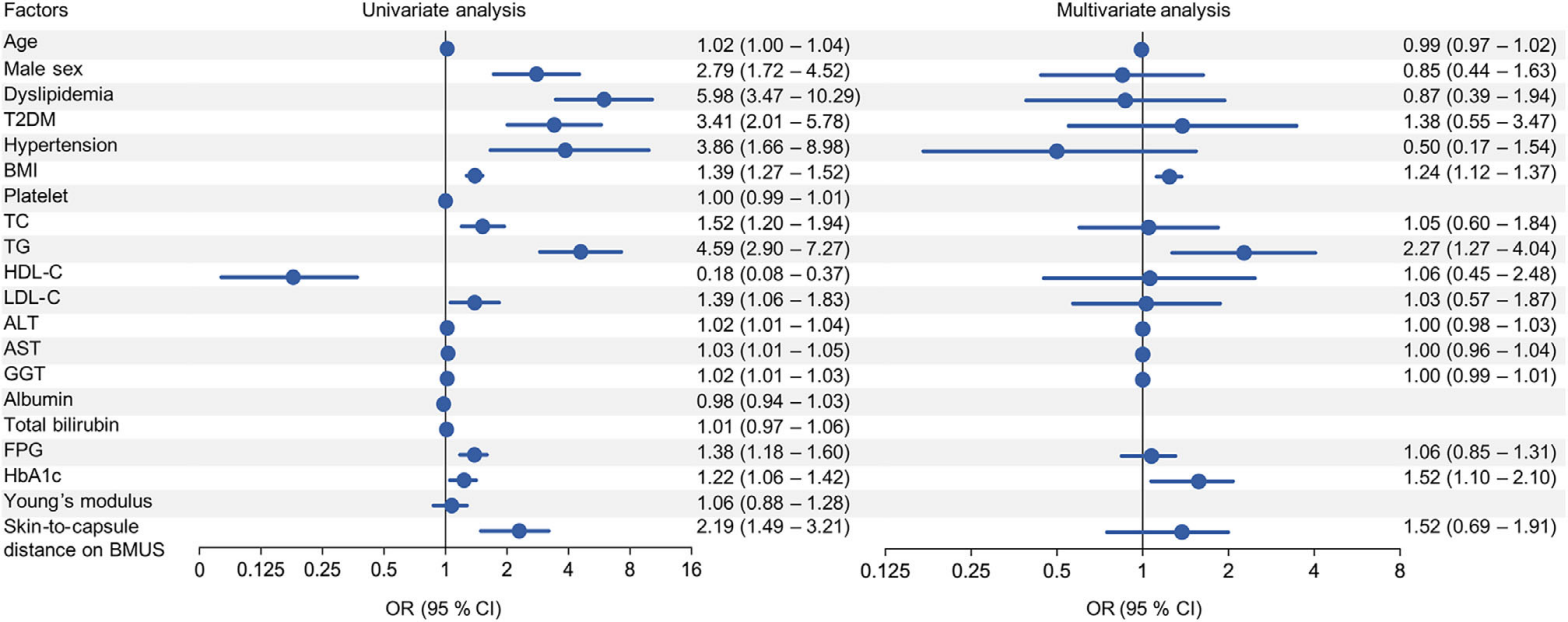
The determinant factors for ultrasound‐derived fat fraction (UDFF) value in the training set. The univariate and multivariate analysis presents odds ratios (ORs) and 95% confidence intervals (CIs) to show the determinant factors for the UDFF values of participants. T2DM, type 2 diabetes mellitus; BMI, body mass index; TC, total cholesterol; TG, triglycerides; HDL‐C, high‐density lipoprotein cholesterol; LDL‐C, low‐density lipoprotein cholesterol; ALT, alanine aminotransferase; AST, aspartate aminotransferase; GGT, gamma‐glutamyl transferase; FPG, fasting plasma glucose; HbA1c, hemoglobin A1c; BMUS, B mode ultrasound.

For the best cutoff value of 7.6% of UDFF measurement to detect hepatic steatosis, the factors associated with UDFF value are shown in Figure 5. By univariate and multivariate analysis, BMI (odd ratio [OR] = 1.24, 95% CI: 1.12 to 1.37, *p* < 0.001), triglyceride (TG) (OR = 2.27, 95% CI: 1.27 to 4.04, *p* = 0.005), and hemoglobin A1c (HbA1c) (OR = 1.52, 95% CI: 1.10 to 2.10, *p* = 0.011) were determinant factors associated with UDFF value.

To determine the implementation of UDFF measurement, the receiver operating characteristics (ROCs) of BMI, TG, and HbA1c were analyzed. The cutoff values of BMI, TG, and HbA1c were 25.1 kg/m^2^ (sensitivity = 73.3%, specificity = 82.3%), 1.2 mmol/L (sensitivity = 80.7%, specificity = 69.0%), and 5.6% (sensitivity = 66.3%, specificity = 76.1%), respectively.

### 2.6 Reliability of UDFF Measurement

For evaluating the validity indicator of UDFF, the average values of IQR of UDFF and IQR/median of UDFF were assessed (1.3 [IQR: 0.50–0.25] vs. 0.13 [IQR: 0.06–0.22]). UDFF values had a mild positive correlation with the IQR of UDFF (*R* = 0.33, 95% CI: 0.19 to 0.45, *p* < 0.001) (Figure S3A) and had a mild negative correlation with IQR/median of UDFF (*R* = −0.21, 95% CI: −0.34 to 0.07, *p* = 0.004) (Figure S3B). The IQR of UDFF of participants with hepatic steatosis is significantly higher than those without hepatic steatosis (*p* < 0.001), while the IQR/median of UDFF had no significant difference between participants with hepatic steatosis and those without hepatic steatosis (*p* = 0.686) (Table 1).

In a subgroup, to investigate the interobserver agreement in performing UDFF measurements, 139 participants from two sites (center 2 and center 3) received repeated UDFF measurements from a second radiologist (at least 5 years’ experience in liver ultrasound scans). The intraclass correlation coefficient (ICC) of those twice UDFF measurements was 0.96 (95% CI: 0.95 to 0.97, *p* < 0.001) (Figure S4). Specifically, the interobserver agreements of UDFF measurements were both excellent in center 2 (ICC = 0.97, 95% CI: 0.95 to 0.98, *p* < 0.001) and center 3 (ICC = 0.94, 95% CI: 0.82 to 0.98, *p* < 0.001).

## 3 Discussion

The prevalence of MASLD is increasing in the global population. However, the detection of hepatic steatosis is not routinely performed in clinical practice as the cost‐benefit uncertainties in diagnostic approach [18]. Quantitative ultrasound methods have been designed to assess hepatic steatosis [19]. Furthermore, developing an ultrasound‐based method to display fat content results as a percentage is recommended to improve clinical practice [20]. UDFF measurement, based on the attenuation coefficient and backscatter coefficient, is designed to evaluate ultrasonic echogenicity and has the potential to assess liver fat content [21], which has the same unit as MRI‐PDFF. In this multicenter study, we prospectively evaluated the diagnostic performance of UDFF measurement to assess hepatic steatosis, with the MRI‐PDFF as the reference standard.

Our study results demonstrated that UDFF showed a strong correlation with the MRI‐PDFF (Spearman, *R* = 0.80, *p* < 0.001) and demonstrated excellent diagnostic performance for assessing hepatic steatosis (AUC = 0.90), which is similar to the result of the previous study of UDFF, using the MRI‐PDFF ≥ 5% as the referent standard [14]. Another study designed to characterize the agreement between UDFF and MRI‐PDFF measurements had an AUC of UDFF of 0.90 for MRI‐PDFF ≥ 5.5% [16]. In addition, the excellent diagnostic performance of the UDFF measurement could be attributed to the use of radiofrequency data, which can provide more information about liver tissue composition and can be lost during BMUS generation [6]. A recent study [13] validated that UDFF (taking the cutoff of 5%) had a higher diagnostic performance than BMUS for detecting hepatic steatosis using MRI‐PDFF ≥ 5% as the reference method. Although the excellent performance of UDFF measurement indicates that it holds promise as a noninvasive and accurate screening method for hepatic fat quantification, it is difficult to directly compare those results because of the differences in the participant samples and the reference.

UDFF value is potentially helpful for assessing hepatic steatosis. With the best cutoff value of 7.6%, the AUCs of UDFF for diagnosing hepatic steatosis were 0.90 in the training set (sensitivity was 87.4%, and specificity was 80.9%) and 0.81 in the validation set (sensitivity was 88.6%, and specificity was 73.3%). However, taking the cutoff of 5% (the same threshold as MRI‐PDFF) for assessing hepatic steatosis, UDFF had a sensitivity of 93.2% and a specificity of 60.9%. These results had a slight discrepancy with previous research, in which UDFF (taking the cutoff of 5%) had a sensitivity of 71.7% and a specificity of 77.6% for detecting hepatic steatosis [13]. Overestimation might exist as there was a low specificity when using 5% as the cutoff value of UDFF.

Radiofrequency ultrasound is less dependent on system settings (such as gain, dynamic range, and filtering) and has minimal operator dependency. All participants in the present study were asked to fast and were in the same position and respiratory phase under the ultrasound examination. The fixed depth and size of ROIs were placed when performing the UDFF measurement, which met the standardized protocol suggested by Ferraioli G et al. [22] and potentially contributed to the reliability of UDFF measurements.

The protocol of UDFF measurement is not standardized [20]. To evaluate the diagnostic performance of UDFF in hepatic steatosis, previous studies performed UDFF from 4 to 10 times for each liver [13, 16, 23]. In this study, UDFF examination was acquired in segments VI/VIII from intercostal and involved 6 measurements for each participant. All UDFF measurements were successful, and the quality indicator (IQR/median of UDFF) had no significant difference between participants with hepatic steatosis and those without hepatic steatosis. UDFF measurement retains the advantages of BMUS, including real‐time image, radiation‐free, and high accessibility. Moreover, UDFF measurement is easy to perform and provides immediate results, which is time‐efficient and inexpensive compared to MRI‐PDFF. In addition, UDFF can be performed simultaneously with liver stiffness measurement in the same plane of liver, making it possible to assess both steatosis and fibrosis.

Several limitations of this study should be noted. First, as the participants underwent MRI‐PDFF measurements rather than liver biopsies, the correlation between the exact amount of hepatic steatosis on pathology and UDFF was not assessed. Second, six different MRI devices at two different field strengths (1.5‐T and 3.0‐T) were used in the study. However, MRI‐PDFF pulse sequences used for our study were the commercially available pulse sequences provided by manufacturers, and the agreement of hepatic MRI‐PDFF measurements among imager manufacturers had been reported previously [24]. Third, although the participants did not receive repeated UDFF measurements at different times, the UDFF measurements were performed by trained radiologists with at least 10 years of experience in liver ultrasound scans. To ensure reliable UDFF values were obtained in the measurement, 6 UDFF acquisitions were obtained for each participant, and the low IQR/median of UDFF could reflect the consistent results from the 6 acquisitions. Fourth, when using the cutoff UDFF value of ≥ 15.9% to diagnose moderate hepatic steatosis, the AUC of UDFF was 0.66 in the validation set, which was lower than that in the training set. The potential reason might be that the median UDFF value of participants with moderate hepatic steatosis was slightly higher than that of participants with mild hepatic steatosis in the validation set. In addition, all participants were ethnic Chinese, and approximately half were overweight or obese. The diagnostic performance of the UDFF technique should be validated in obese populations. Finally, as the prevalence of T2DM was 39.0% in our study, further studies might be needed to validate these results in a diabetes clinic.

## 4 Conclusions

UDFF has excellent diagnostic performance in detecting and grading hepatic steatosis using MRI‐PDFF as the reference standard and can be potentially used in clinical practice.

## 5 Materials and Methods

### 5.1 Study Designs

This prospective study was approved by the institutional review board (XHEC‐C‐2022‐121‐1) and registered at ClinicalTrials.gov (NCT05802199). Informed consent was obtained from all participants in the study. The principle of the Declaration of Helsinki was followed.

This multicenter cohort study enrolled participants referred for assessment of hepatic steatosis via BMUS from eight tertiary hospitals in China (Xinhua Hospital Affiliated to Shanghai Jiao Tong University School of Medicine; Zhongda Hospital, Medical School, Southeast University; Affiliated Hangzhou First People's Hospital, Zhejiang University School of Medicine; Lishui People's Hospital; Shandong Public Health Clinical Center; the First Affiliated Hospital of Nanjing Medical University; the Second Affiliated Hospital of Anhui Medical University; and Bozhou Hospital Affiliated to Anhui Medical University). The inclusion criteria were as follows: (a) participants over 18 years old with or suspected steatotic liver disease; (b) participants planned to undergo UDFF and MRI‐PDFF measurements, performed in either order within 1 week; and (c) participants who had complete clinical and laboratory data recorded. The exclusion criteria were as follows: (a) substantial alcohol consumption [25]; (b) clinical, laboratory, or pathological evidence of other liver diseases (such as viral hepatitis, autoimmune hepatitis, or alpha‐1‐antitrypsin deficiency); (c) participants who failed to obtain valid MRI‐PDFF images due to the motion artifacts or exceeding bore diameter; and (d) participants who were uncooperative to obtain valid UDFF images.

### 5.2 Ultrasound Examination and UDFF Measurement

All ultrasound examinations were performed using an Acuson Sequoia ultrasound system (Siemens Healthineers), equipped with a special deep abdominal transducer (DAX: 1.0–5.7 MHz) by one of eight radiologists (at least 10 years’ experience in liver ultrasound scans) who were blinded to participants’ clinical findings and other imaging results.

The participants were asked to fast for 6 h before the ultrasound examination. For the procedure, participants were in the dorsal decubitus position with maximal right arm abduction. A breath‐holding maneuver (holding a breath during a calm breathing cycle) was applied to avoid motion artifacts of ultrasound. First, liver morphology, parenchyma echogenicity, and skin‐to‐capsule distance were evaluated by BMUS.

The UDFF measurements were performed immediately after the BMUS examination, using the same ultrasound system and transducer by the same radiologist. A fixed‐size region of interest (ROI) was positioned in the liver parenchyma 1.5 cm below the capsule. For each participant, UDFF examination was acquired in segments VI/VIII from intercostal and involved 6 measurements, and the median UDFF value was taken as the estimate for hepatic steatosis. A valid UDFF value was displayed as a percentage. Invalid UDFF measurement for one participant is considered if all 6 UDFF acquisitions were not displayed. The UDFF images were stored. The time spent obtaining 6 UDFF acquisitions per participant was recorded in minutes.

Simultaneously, during UDFF measurements, SWV (in m/s) and Young's modulus (in kPa) were automatically measured when the auto point shear wave elastography mode was switched on. Six acquisitions of SWV and Young's modulus were recorded for each participant, and their median values were calculated to estimate liver stiffness.

### 5.3 MRI‐PDFF Examination and Imaging Analysis

Participants were examined with contemporaneous chemical shift‐encoded liver MRI using 3.0‐T systems (Vida and Verio, Siemens Healthcare; Ingenia, Philips; Discovery, GE Healthcare) or 1.5‐T systems (Signa HD, GE Healthcare; uMR660, UNITED IMAGING). MRI‐PDFF pulse sequences used for this study are the commercially available pulse sequences provided by manufacturers. MRI‐PDFF is the ratio of MRI‐visible fat protons to the sum of MRI‐visible fat and bulk (free) water protons [26]. The multiecho source images were recorded offline for analysis. MRI‐PDFF maps were generated pixel by pixel from the source images. The MRI‐PDFF maps were automatically generated by a custom algorithm. This algorithm simultaneously estimates T2* and PDFF by considering the multifrequency interference of protons in hepatic fat [27, 28].

Another eight radiologists (at least 10 years’ experience) in the departments of radiology were blinded to participants’ clinical data and ultrasound results manually placed circular ROIs (radius of 1 cm) in each of the nine Couinaud hepatic segments at least 1.5 cm under the liver capsule, avoiding artifacts and major blood vessels [7]. The MRI‐PDFF (in percentage) in each of the nine ROIs was recorded, and the value across the entire liver was displayed as the mean MRI‐PDFF values of all nine ROIs [29]. The thresholds of MRI‐PDFF ≥ 5%, ≥ 15%, and ≥ 25% were used as the reference standard for defining mild, moderate, and severe hepatic steatosis, respectively [30].

### 5.4 Statistical Analysis

Quantitative variables were summarized as median (IQR) or mean ± standard deviation, as appropriate. Qualitative variables were presented as absolute (number, *n*) and relative (percentage,%). The Student *t* test, chi‐squared, Fisher's exact test, or Mann–Whitney *U* test was used in the univariable comparisons. The Jonckheere–Terpstra test was performed to compare multiple groups. Linearity and bias were evaluated by plotting the data in Bland–Altman plots, bar plots, and scatterplots. Pearson's or Spearman's correlation coefficients were used to assess correlation. The primary cohort was used as a training set. This study was later externally validated in participants meeting the same inclusion and exclusion criteria from one of the eight centers. The ROC was calculated, and the AUC was performed to assess the diagnostic performance of UDFF measurements for hepatic steatosis. The Youden index (sensitivity + specificity − 1) was calculated to identify the best cutoff values of UDFF for diagnosing and grading hepatic steatosis in the training set. The AUCs, sensitivity, and specificity of the predetermined cutoff values were calculated in the validation set. Independent variables with significance *p* < 0.05 in univariate analysis were introduced to multivariate analysis to identify the determinant factors. OR and 95% CIs were estimated. In addition, to determine the implementation of UDFF measurement, the ROCs of the determinant factors of UDFF values were analyzed. ICC with 95% CI was calculated to assess the agreement of radiologists in subgroup analysis using the two‐way random model. All statistical analyses were performed with SPSS 29.0 (IBM, Armonk, NY) and GraphPad Prism 10.2.0 (GraphPad Software Inc., USA). Two‐tailed *p* < 0.05 was considered statistically significant.

## Author Contributions

Y.D., C.F.D., and X.Q. were responsible for the study concept. Y.H., J.L., C.L., D.S., C.Z., Y.R., J.L., L.J., R.S., H.W., Z.W., S.L., F.J., X.X., L.L., R.Q., P.R., C.S., W.Y., Y.G., J.C., and Y.W. were responsible for the data collection and interpretation. Y.H., J.L., C.L., D.S., C.Z., Y.R., and J.L. contributed to data analysis and writing the manuscript draft. Y.H., J.L., C.L., D.S., C.Z., Y.R., J.L., V.W.S.W., W.W., WK.S., Y.D., C.F.D., and X.Q. were responsible for the critical review of manuscript. All authors have read and approved the final manuscript.

## Ethics Statement

This study was approved by the institutional review board (XHEC‐C‐2022‐121‐1) and registered at ClinicalTrials.gov (NCT05802199). Informed consent was obtained from all participants in the study.

## Conflicts of Interest

All authors declared no conflict of interest.

## Supporting information



Supporting Information

## Data Availability

The data that support the findings of this study are available from the corresponding author upon reasonable request.
