# Supplementary material for: Noninvasive Quantification of Hepatic Steatosis Using Ultrasound‐Derived Fat Fraction (CHESS2303): A Prospective Multicenter Study
Source: MedComm (2020). 2025 Feb 27;6(3):e70123. doi: 10.1002/mco2.70123 (PMC11868440; doi:10.1002/mco2.70123)
Supplement: Supplementary file 1 — Supporting Information [file MCO2-6-e70123-s001.docx]

**Noninvasive Quantification of Hepatic Steatosis Using Ultrasound-Derived Fat Fraction (CHESS2303): A Prospective Multicenter Study**

# Running Head

Quantification of hepatic steatosis using UDFF

# Authors

Yunlin Huang^1,#^, Jia Li^2,#^, Chuan Liu^3,4,#^, Danlei Song^2,#^, Chuanlong Zhu^5,#^, Yongfeng Ren^6,#^, Jiaojian Lv^7,#^, Longfeng Jiang^5^, Rong Shan^8^, Hao Wang^8^, Zhou Wang^6^, Siqin Long^7^, Fan Jiang^9^, Xiang Xie^10^, Liren Lu^11^, Ruixiang Qi^11^, Pengfei Rong^12^, Chuxiao Shao^13^, Wang Yao^14^, Youfang Gao^15^, Wenping Wang^16^, Juan Cheng^1^, Vincent Wai-Sun Wong^17^, Ying Wang^1^, Wai-Kay Seto^18^, Yi Dong^1,*^, Christoph F. Dietrich^19,*^, Xiaolong Qi^3,4,*^

^#^ Share the co-first authorship

^*^ Share the co-corresponding authorship

# Affiliations

^1^ Department of Ultrasound, Xinhua Hospital Affiliated to Shanghai Jiao Tong University School of Medicine, Shanghai, China

^2^ Department of Ultrasound, Zhongda Hospital, Medical School, Southeast University, Nanjing, China

^3^ Liver Disease Center of Integrated Traditional Chinese and Western Medicine, Department of Radiology, Zhongda Hospital, Medical School, Southeast University, Nurturing Center of Jiangsu Province for State Laboratory of AI Imaging & Interventional Radiology (Southeast University), Nanjing, China

^4^ Basic Medicine Research and Innovation Center of Ministry of Education, Zhongda Hospital, Southeast University; State Key Laboratory of Digital Medical Engineering, Nanjing, China

^5^ Department of Infectious Diseases, The First Affiliated Hospital of Nanjing Medical University, Nanjing, China

^6^ Department of Ultrasound, Bozhou Hospital Affiliated to Anhui Medical University, Bozhou, China

^7^ Department of Liver Disease, Lishui People’s Hospital, Lishui, China

^8^ Department of Ultrasound, Shandong Public Health Clinical Center, Shandong University, Shandong, China

^9^ Department of Ultrasound Medicine, the Second Affiliated Hospital of Anhui Medical University, Hefei, China

^10^ Department of Interventional Therapy, The Second Affiliated Hospital of Anhui Medical University, Hefei, China

^11^ Department of Ultrasound, Affiliated Hangzhou First People’s Hospital, Zhejiang University School of Medicine, Hangzhou, China

^12^ Department of Radiology, The Third Xiangya Hospital of Central South University, Changsha, China

^13^ Key Laboratory of Joint Diagnosis and Treatment of Chronic Liver Disease and Liver Cancer of Lishui, Lishui People’s Hospital, Lishui, China

^14^ Department of Ultrasound, Lishui People’s Hospital, Lishui, China

^15^ Department of Infectious Disease, The People’s Hospital of Bozhou, Bozhou, China

^16^ Department of Ultrasound, Zhongshan Hospital, Fudan University, Shanghai, China

^17^ Department of Medicine and Therapeutics, The Chinese University of Hong Kong, Hong Kong, China

^18^ Department of Medicine, Queen Mary Hospital, The University of Hong Kong, Hong Kong, China

^19^ Department of Allgemeine Innere Medizin, Kliniken Hirslanden Beau Site, Salem Und Permanence, Bern, Switzerland

# Correspondence

Dr. Xiaolong Qi, MD

Chair, Liver Health Consortium in China (CHESS)

Liver Disease Center of Integrated Traditional Chinese and Western Medicine, Department of Radiology, Zhongda Hospital, Medical School, Southeast University, Nurturing Center of Jiangsu Province for State Laboratory of AI Imaging & Interventional Radiology (Southeast University), Nanjing, China; Basic Medicine Research and Innovation Center of Ministry of Education, Zhongda Hospital, Southeast University; State Key Laboratory of Digital Medical Engineering, Nanjing, China

Email: qixiaolong@vip.163.com

Dr. Christoph F. Dietrich, MD, Ph.D, MBA

Department of Allgemeine Innere Medizin, Kliniken Hirslanden Beau Site, Salem Und Permanence, Schänzlihalde 11, Bern, 3036, Switzerland

Email: c.f.dietrich@googlemail.com

Dr. Yi Dong, MD

Department of Ultrasound, Xinhua Hospital Affiliated to Shanghai Jiao Tong University School of Medicine, 1665 Kongjiang Road, Shanghai, 200092, China

Email: [drdaisydong@hotmail.com](mailto:drdaisydong@hotmail.com)

Supplementary Materials -- Tables

Table S1. Characteristics of participants from each center.

| **Characteristics** | **Center 1 (n = 42)** | **Center 2 (n = 125)** | **Center 3 (n = 14)** | **Center 4 (n = 20)** | **Center 5 (n = 25)** | **Center 6 (n = 30)** | **Center 7 (n = 20)** | **Center 8 (n = 24)** | ***P* value** |
| --- | --- | --- | --- | --- | --- | --- | --- | --- | --- |
| **Demographic data** | | | | | | | | | |
| Age (years) | 40.0 (37.8 – 50.3) | 37.0 (27.5 – 51.5) | 36.5 (25.5 – 45.0) | 53.0 (34.0 – 57.0) | 49.0 (43.5 – 54.5) | 31.5 (25.0 – 36.8) | 45.5 (33.3 – 52.8) | 38.0 (36.0 – 48.5) | 0.851 |
| Sex |  |  |  |  |  |  |  |  | 0.418 |
| Male | 22 (52.4) | 66 (52.8) | 4 (28.6) | 6 (30.0) | 7 (28.0) | 13 (43.3) | 17 (85.0) | 18 (75.0) |  |
| Female | 20 (47.6) | 59 (47.2) | 10 (71.4) | 14 (70.0) | 18 (72.0) | 17 (56.7) | 3 (15.0) | 6 (25.0) |  |
| BMI (kg/m^2^) | 27.7 (23.4 – 29.2) | 24.2 (21.5 – 27.1) | 26.0 (22.9 – 29.8) | 24.9 (24.1 – 27.6) | 25.1 (22.7 – 26.3) | 23.6 (21.7 – 27.7) | 26.9 (24.3 – 30.6) | 28.2 (26.1- 30.0) | 0.139 |
| **Metabolic factors** |  |  |  |  |  |  |  |  |  |
| Dyslipidemia | 19 (45.2) | 52 (41.6) | 7 (50.0) | 8 (40.0) | 9 (36.0) | 11 (36.7) | 18 (90.0) | 12 (50.0) | 0.166 |
| T2DM | 5 (11.9) | 70 (56.0) | 2 (14.3) | 3 (15.0) | 7 (28.0) | 0 (0) | 13 (65.0) | 17 (70.8) | 0.363 |
| Hypertension | 0 (0) | 17 (13.6) | 2 (14.3) | 6 (30.0) | 8 (32.0) | 0 (0) | 8 (40.0) | 4 (16.7) | 0.003 |
| Obesity | 9 (21.4) | 17 (13.6) | 3 (21.4) | 3 (15.0) | 2 (8.0) | 3 (10.0) | 7 (35.0) | 6 (25.0) | 0.576 |
| **Biochemical profile** | | | | | | | | | |
| Platelet (10^9^/L) | 259.0  (222.0 – 308.8) | 202.0  (178.5 – 245.0) | 229.5  (198.5 – 253.3) | 244.5  (208.3 – 306.3.5) | 248.0  (199.5 – 285.5) | 251.5  (216.5 – 305.0) | 207.0  (182.5 – 238.0) | 264.0  (214.3 – 301.5) | 0.282 |
| TC (mmol/L) | 4.8 (4.0 – 5.8) | 4.4 (3.8 – 5.5) | 4.8 (4.5 – 5.6) | 5.3 (4.7 – 5.8) | 4.7 (4.1 – 5.6) | 4.8 (4.2 – 5.4) | 4.9 (4.2 – 5.6) | 5.2 (4.5 – 5.6) | 0.040 |
| TG (mmol/L) | 1.4 (1.0 – 1.7) | 1.3 (0.8 – 2.2) | 1.3 (1.0 – 1.8) | 1.5 (1.1 – 2.2) | 1.0 (0.7 – 2.0) | 1.3 (1.0 – 1.9) | 2.9 (2.0 – 3.6) | 1.6 (1.2 – 2.9) | 0.005 |
| HDL-C (mmol/L) | 1.3 (1.0 – 1.4) | 1.3 (1.0 – 1.6) | 1.2 (1.0 – 1.4) | 1.4 (1.1 – 1.6) | 1.4 (1.1 – 1.6) | 1.2 (1.0 – 1.2) | 1.0 (0.9 – 1.2) | 1.4 (1.1 – 1.5) | 0.507 |
| LDL-C (mmol/L) | 3.3 (2.5 – 4.2) | 2.5 (2.0 – 3.2) | 3.0 (2.5 – 3.6) | 3.1 (2.5 – 3.3) | 2.8 (2.4 – 3.5) | 3.0 (2.5 – 3.6) | 3.0 (2.2 – 3.4) | 3.3 (2.9 – 3.7) | 0.231 |
| ALT (IU/L) | 27.0 (21.8 – 40.5) | 21.0 (13.6 – 34.0) | 54.0 (22.0 – 85.8) | 30.0 (17.8 – 62.5) | 17.0 (11.5 – 24.0) | 21.2 (14.5 – 37.4) | 35.5 (23.3 – 55.0) | 26.0 (21.0 – 34.8) | 0.507 |
| AST (IU/L) | 22.1 (20.0 – 27.8) | 19.0 (16.0 – 25.0) | 47.5 (20.0 – 60.3) | 26.5 (22.3 – 41.5) | 20.0 (14.0 – 25.0) | 21.5 (18.6 – 26.1) | 24.0 (19.3 – 32.0) | 23.5 (20.3 – 27.8) | 0.112 |
| GGT (IU/L) | 35.0 (19.8 – 53.5) | 23.0 (14.0 – 44.5) | 43.0 (21.5 – 91.0) | 31.0 (14.5 – 45.3) | 25.0 (12.5 – 44.5) | 22.2 (17.9 – 45.1) | 34.5 (25.8 – 69.5) | 36.5 (24.3 – 61.8) | 0.104 |
| Albumin (g/L) | 44.9 (43.9 – 46.8) | 44.6 (42.7 – 47.6) | 44.3 (43.1 – 45.5) | 44.5 (44.0 – 45.9) | 48.9 (46.5 – 51.4) | 45.2 (43.6 – 47.3) | 41.1 (38.4 – 44.0) | 46.0 (44.5 – 48.0) | 0.431 |
| Total bilirubin (μmol/L) | 9.9 (8.6 – 10.7) | 10.0 (7.3 – 11.7) | 11.1 (8.3 – 14.9) | 11.1 (9.0 – 14.6) | 13.6 (11.1 – 17.6) | 11.3 (9.1 – 15.0) | 13.9 (12.1 – 16.1) | 15.3 (10.0 – 18.7) | < 0.001 |
| FPG (mmol/L) | 5.4 (4.9 – 5.7) | 6.0 (5.0 – 9.6) | 5.3 (4.9 – 5.9) | 5.1 (5.0 – 5.6) | 5.1 (4.9 – 6.0) | 4.6 (4.3 – 4.8) | 5.2 (4.6 – 9.6) | 5.8 (5.6 – 6.0) | 0.017 |
| HbA1c (%) | 5.6 (5.3 – 6.0) | 5.7 (4.9 – 8.4) | 5.6 (5.2 – 6.3) | 5.4 (5.2 – 5.7) | 5.7 (5.5 – 6.4) | 5.3 (5.1 – 5.5) | 6.4 (5.7 – 9.3) | 5.5 (5.4 – 5.8) | 0.936 |
| **Ultrasonic data** |  |  |  |  |  |  |  |  |  |
| Median of UDFF (%) | 12.5 (7.6 – 19.4) | 9.2 (4.3 – 19.0) | 13.1 (6.1 – 22.0) | 9.3 (7.1 – 15.1) | 5.8 (4.0 – 10.4) | 5.3 (3.8 – 8.4) | 17.1 (10.2 – 22.0) | 13.6 (7.4 – 19.4) | 0.405 |
| IQR of UDFF (%) | 3.2 (1.5 – 5.5) | 1.0 (0.3 – 1.8) | 1.4 (0.7 – 3.2) | 1.5 (0.6 – 2.7) | 1.3 (0.7 – 2.4) | 1.0 (0 – 1.9) | 1.8 (1.0 – 2.2) | 1.4 (0.9 – 2.5) | 0.095 |
| IQR/Median of UDFF | 0.25 (0.13 – 0.48) | 0.10 (0.01 – 0.17) | 0.13 (0.08 – 0.20) | 0.16 (0.06 – 0.23) | 0.18 (0.12 – 0.24) | 0.17 (0 – 0.28) | 0.10 (0.06 – 0.18) | 0.12 (0.06 – 0.19) | 0.088 |
| SWV (m/s) | 1.23 (1.12 – 1.41) | 1.05 (0.98 – 1.15) | 1.10 (0.99 – 1.26) | 1.17 (1.07 – 1.29) | 1.11 (1.03 – 1.18) | 1.14 (1.09 – 1.30) | 1.18 (1.10 – 1.33) | 1.17 (1.10 – 1.31) | 0.106 |
| Young’s modulus (kPa) | 4.56 (3.73 – 6.39) | 3.52 (3.02 – 4.02) | 3.73 (3.0 – 4.99) | 4.04 (3.35 – 4.90) | 3.82 (3.24 – 4.31) | 3.88 (3.58 – 5.12) | 4.12 (3.62 – 5.15) | 4.12 (3.68 – 5.15) | 0.317 |
| Skin-to-capsule distance on BMUS (cm) | 2.6 (2.3 – 3.0) | 1.9 (1.6 – 2.2) | 2.6 (2.0 – 2.9) | 2.0 (1.7 – 2.3) | 1.9 (1.6 – 2.1) | 3.6 (3.1 – 4.0) | 2.3 (2.1 – 3.3) | 2.3 (2.0 – 2.6) | 0.014 |
| **MRI-PDFF (%)** | 8.1 (4.8 – 12.5) | 7.0 (3.8 – 13.8) | 14.0 (8.0 – 24.6) | 8.0 (4.0 – 10.6) | 3.6 (2.5 – 7.3) | 3.1 (1.1 – 6.5) | 11.6 (6.8 – 17.6) | 10.0 (6.5 – 14.0) | 0.488 |
| *Note*: Quantitative variables were presented as median (interquartile range); Qualitative variables were presented as absolute (number), and data in parentheses are the percentage (%).  Abbreviations: BMI, body mass index; T2DM, type 2 diabetes mellitus; TC, total cholesterol; TG, triglycerides; HDL-C, high-density lipoprotein cholesterol; LDL-C, low-density lipoprotein cholesterol; ALT, alanine aminotransferase; AST, aspartate aminotransferase; GGT, gamma-glutamyl transferase; FPG, fasting plasma glucose; HbA1c, hemoglobin A1c; UDFF, ultrasound-derived fat fraction; IQR, interquartile range; SWV, shear wave velocity; BMUS, B mode ultrasound. | | | | | | | | | |

Supplementary Materials – Figures

**Figure S1.** The determinant factors associated with hepatic steatosis in the training set. The univariate and multivariate analysis presents odds ratios (OR) and 95% confidence intervals (CIs) to show the determinant factors for participants with hepatic steatosis. T2DM, type 2 diabetes mellitus; BMI, body mass index; TC, total cholesterol; TG, triglycerides; HDL-C, high-density lipoprotein cholesterol; LDL-C, low-density lipoprotein cholesterol; ALT, alanine aminotransferase; AST, aspartate aminotransferase; GGT, gamma-glutamyl transferase; FPG, fasting plasma glucose; HbA1c, hemoglobin A1c; UDFF, ultrasound-derived fat fraction; BMUS, B mode ultrasound.

**
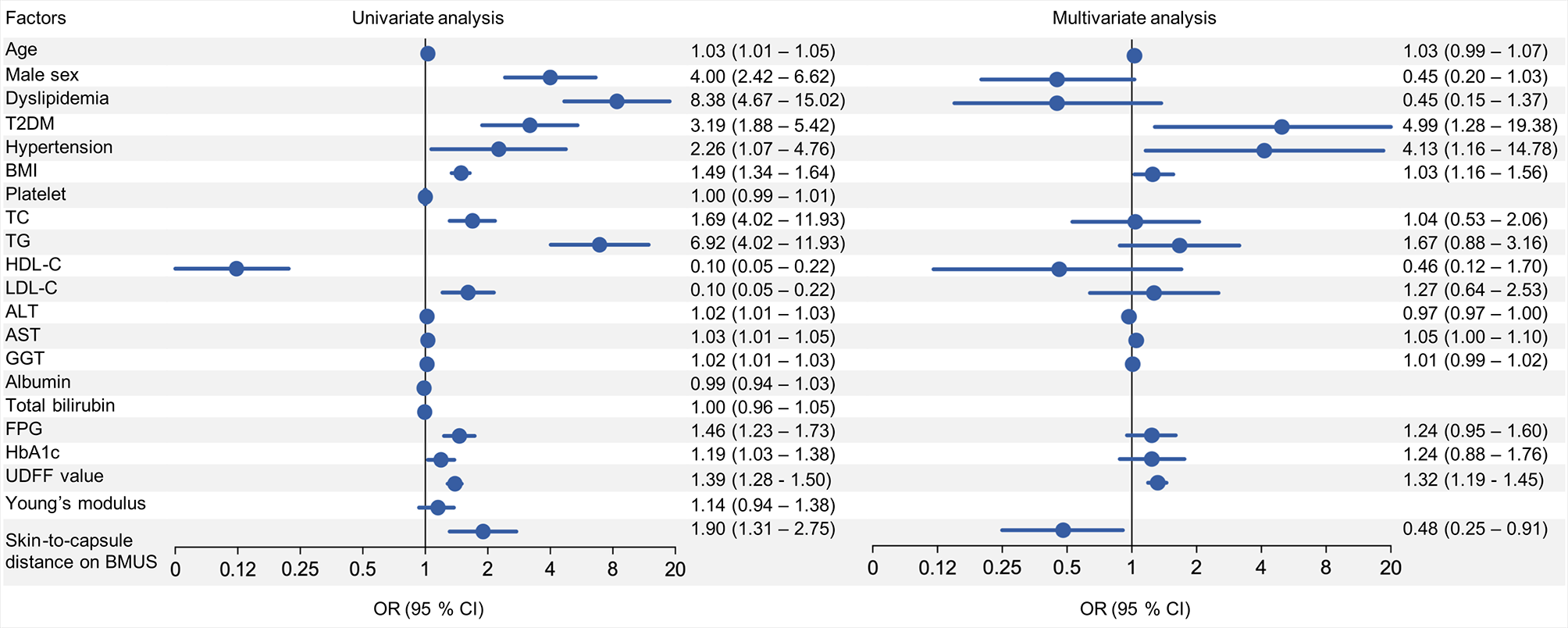
**

**Figure S2.** A bar plot showing the 6 actual individual measurements of ultrasound-derived fat fraction (UDFF) per participant in the training set. Participants are sorted in order of increasing median UDFF values.

**
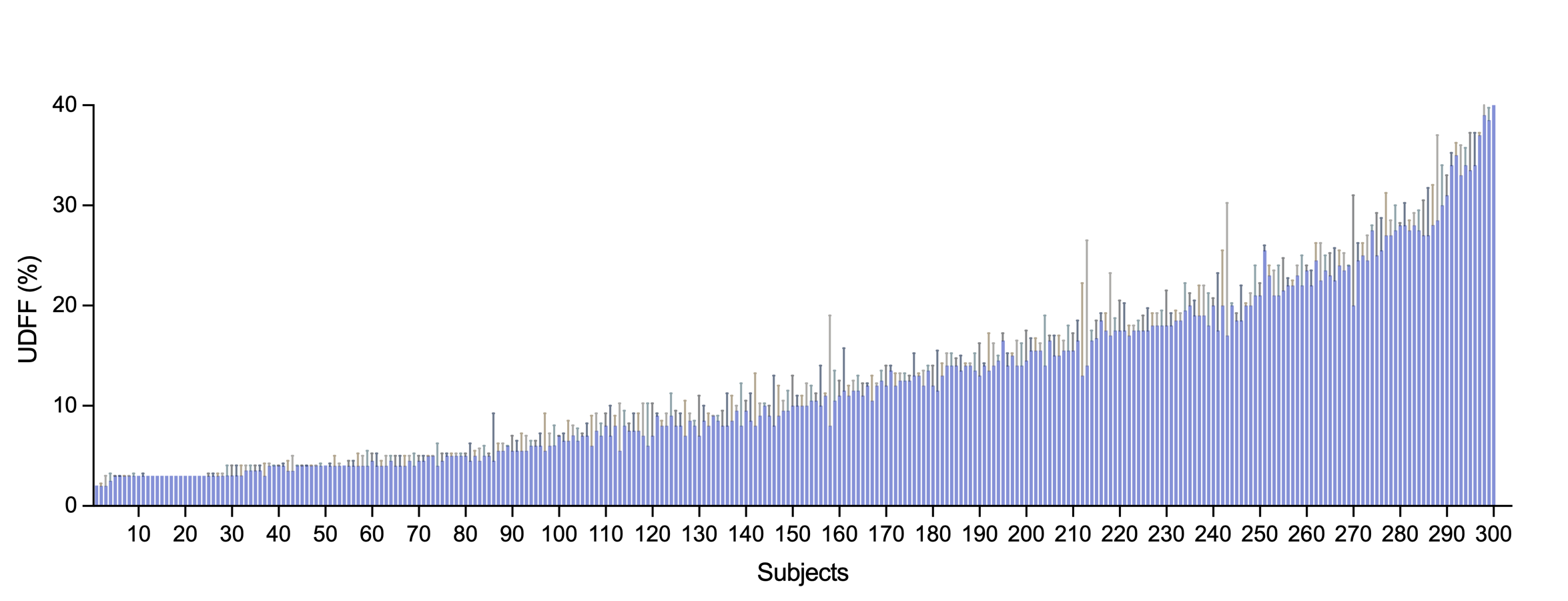
**

**Figure S3.** Scatterplots showing the correlation between ultrasonic parameters. (A) Ultrasound-derived fat fraction (UDFF) and the interquartile range (IQR) of UDFF and (B) the IQR/median of UDFF.

**
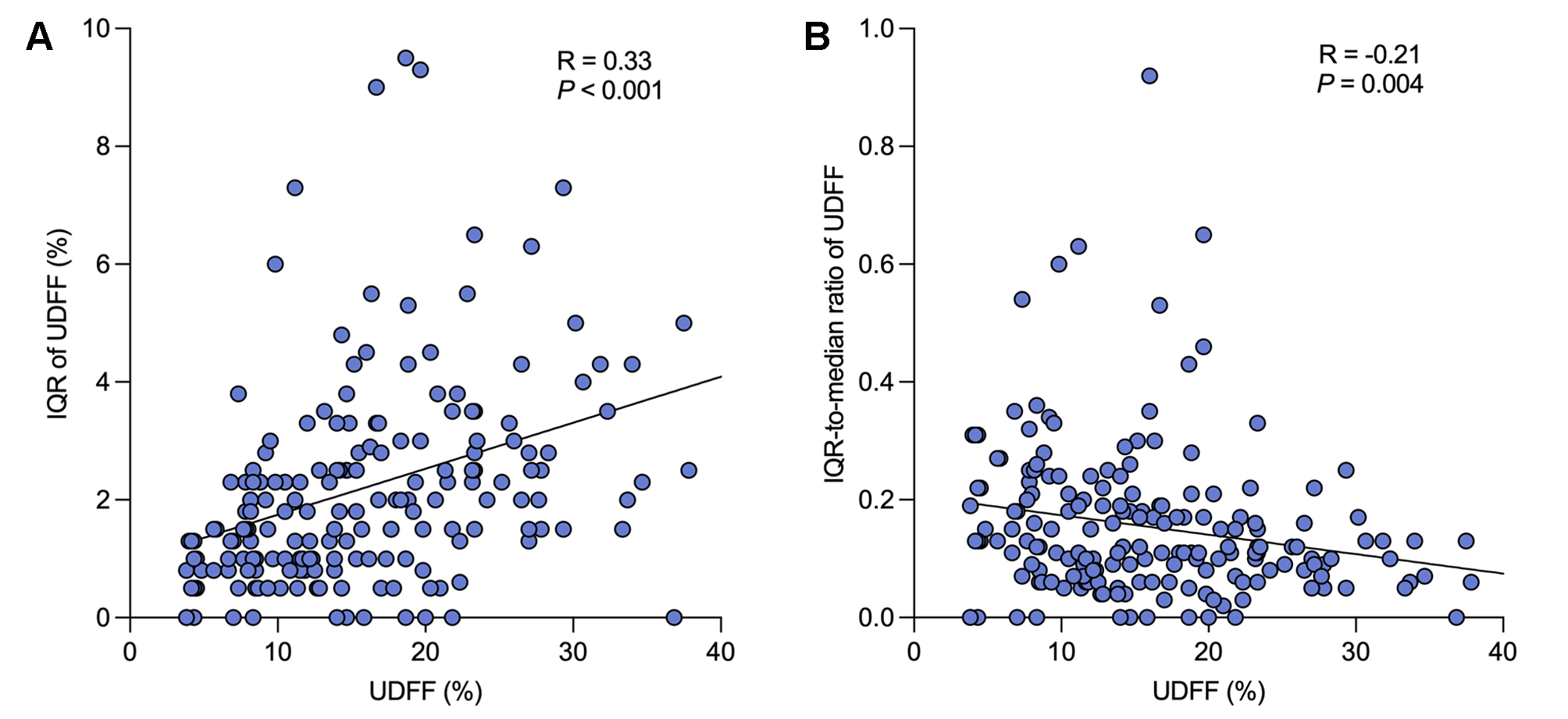
**

Figure S4. Scatterplots of ultrasound-derived fat fraction obtained by two different radiologists, along with the linear regression line. The intraclass correlation coefficient was 0.96 (95 % confidence interval: 0.95 – 0.97, *P* < 0.001).

**
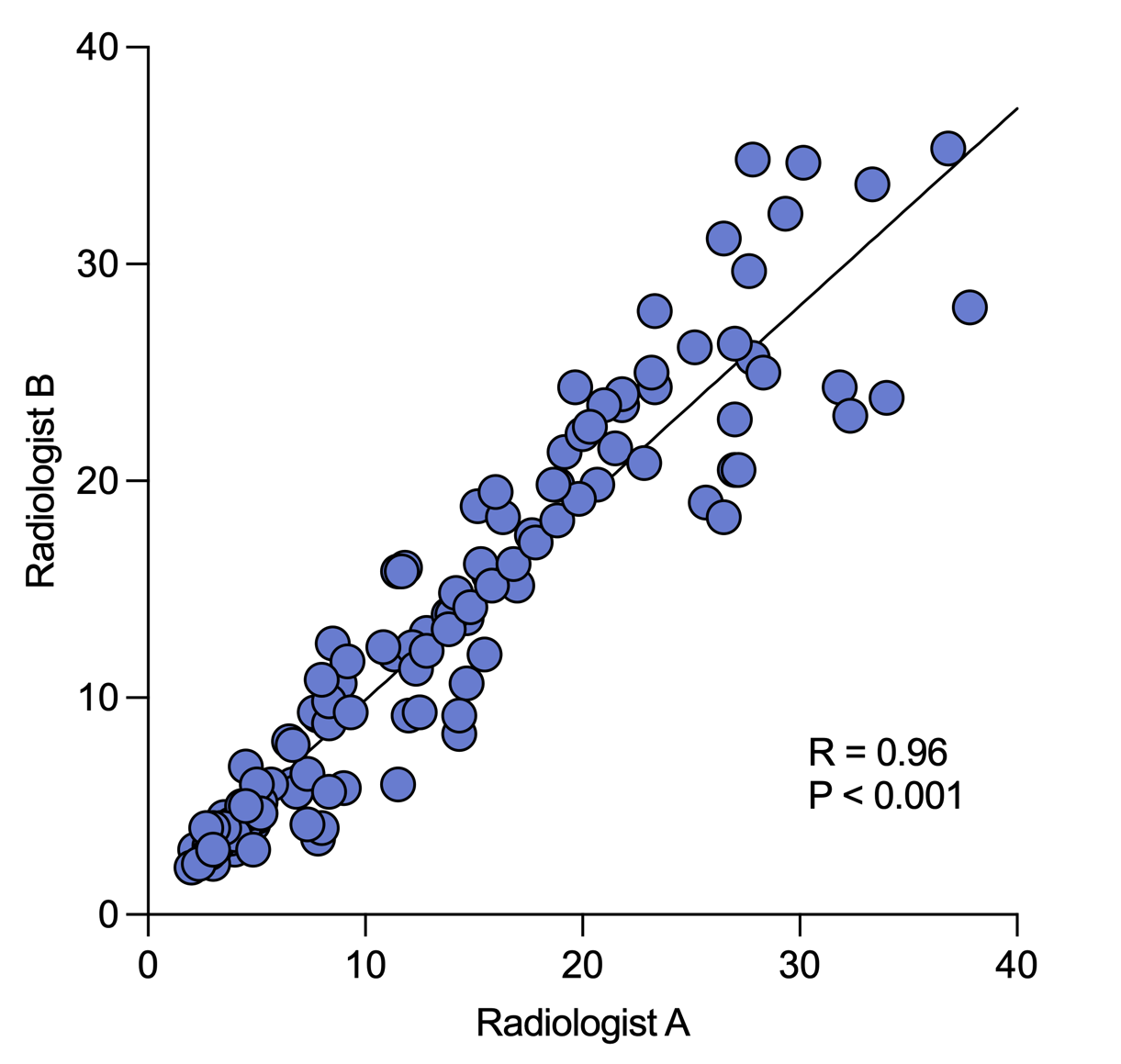
**

**Supplementary Materials – Subgroup Analysis**

**Diagnostic performance of UDFF and CAP in subgroup**

To compare the diagnostic performance of UDFF and CAP, 67 participants in two sites (center 2 and center 3) from the training set received both UDFF and CAP measurements on the same day. The CAP measurements were performed using the FibroScan 502 Touch system (Echosens, Paris, France). The median value of 10 valid CAP measurements was calculated, and the results were presented as dB/m. CAP measurement failure was recorded when no valid value was obtained after at least 10 shots. One participant was too thick of an abdominal subcutaneous fat layer to assess CAP successfully. Finally, 66 participants were included in the subgroup analysis.

Using MRI-PDFF as a reference to detect hepatic steatosis, the AUCs of UDFF and CAP for assessing mild hepatic steatosis (MRI-PDFF ≥ 5 %) were 0.90 (95 % CI: 0.82 – 0.97) and 0.88 (95 % CI: 0.80 – 0.97), respectively. The AUCs of UDFF and CAP for assessing moderate hepatic steatosis (MRI-PDFF ≥ 15 %) were 0.88 (95 % CI: 0.79 – 0.98) and 0.82 (95 % CI: 0.64 – 0.99), respectively. The AUCs of UDFF and CAP for assessing severe hepatic steatosis (MRI-PDFF ≥ 25 %) were 0.89 (95 % CI: 0.78 – 1.00) and 0.86 (95 % CI: 0.77 – 0.95), respectively (**Figure S5**). However, DeLong’s test showed that there were no significant differences in diagnostic performance between UDFF and CAP measurement for grading mild, moderate, and severe hepatic steatosis (*P* = 0.781, *P* = 0.220, and *P* = 0.530, respectively) (**Table S2**).

**Figure S5.** The diagnostic performance of ultrasound-derived fat fraction (UDFF) and attenuation parameter (CAP) for detecting (A) mild, (B) moderate, and (C) severe hepatic steatosis using magnetic resonance imaging proton density fat fraction ≥ 5 %, ≥ 15 %, and ≥ 25 % as the reference, respectively. AUC, area under the receiver operating characteristic curve.

**
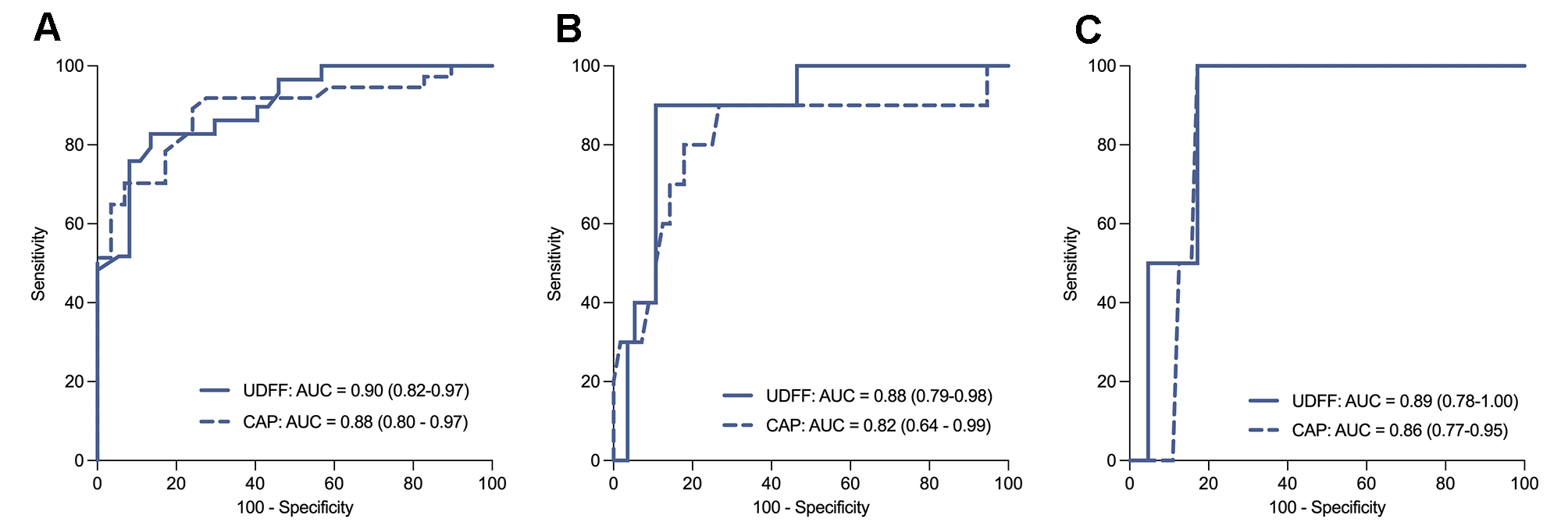
**

**Table S2. Comparison between the performances of UDFF and CAP for detecting hepatic steatosis in 66 participants**

|  | **UDFF** | **CAP** | ***P* value** |
| --- | --- | --- | --- |
| **Mild hepatic steatosis**  **(MRI-PDFF ≥ 5 %)** |  |  |  |
| AUC | 0.90 (0.82 – 0.97) | 0.88 (0.80 – 0.97) | 0.781 |
| Sensitivity (%) | 82.8 (65.5 – 92.4) | 89.2 (75.3 – 95.7) |  |
| Specificity (%) | 86.5 (72.0 – 94.1) | 75.9 (57.9 – 87.8) |  |
| **Moderate hepatic steatosis**  **(MRI-PDFF ≥ 15 %)** |  |  |  |
| AUC | 0.88 (0.79 – 0.98) | 0.82 (0.64 – 0.99) | 0.220 |
| Sensitivity (%) | 90.0 (59.6 – 99.5) | 90.0 (59.6 – 99.5) |  |
| Specificity (%) | 89.3 (78.5 – 95.0) | 73.2 (60.4 – 83.0) |  |
| **Severe hepatic steatosis**  **(MRI-PDFF ≥ 25 %)** |  |  |  |
| AUC | 0.89 (0.78 – 1.00) | 0.86 (0.77 – 0.95) | 0.530 |
| Sensitivity (%) | 100.0 (17.8 – 100.0) | 100.0 (17.8 – 100.0) |  |
| Specificity (%) | 82.8 (71.8 – 90.1) | 82.8 (71.8 – 90.1) |  |
| Abbreviations: UDFF, ultrasound-derived fat fraction; CAP, controlled attenuation parameter; MRI-PDFF, magnetic resonance imaging proton density fat fraction; AUC, area under the receiver operating characteristic curve. | | | |
